# Exploring knowledge, attitude and practices of essential newborn care among puerperal mothers who gave birth at public health facilities of southwest Ethiopia: a mixed study

**DOI:** 10.11604/pamj.2024.49.104.43893

**Published:** 2024-12-02

**Authors:** Endale Tamiru Burayu

**Affiliations:** 1 Department of Midwifery, College of Health Sciences, Mattu University, Mattu, Southwest, Ethiopia

**Keywords:** Knowledge, attitude, practice, essential newborn care, puerperal period, Ilubabor zone

## Abstract

**Introduction:**

essential newborn care guidelines have been developed to cover all aspects of care from conception to postnatal care. There were concerns about knowledge and practices related to newborn care in Ethiopia, and unfortunately, neonatal mortality remained high. To address this issue, we conducted a new study to examine the attitudes, knowledge and practices of postpartum mothers who gave birth in public health facilities in Ilubabor zone, southwest Ethiopia.

**Methods:**

a mixed-institution cross-sectional study was conducted from June 20 to August 20, 2023. Participants were selected using systematic random sampling until the sample size was 381. Data were collected through semi-structured questionnaires and in-depth interviews. Data were then entered into Epidata and analyzed using SPSS.

**Results:**

of the postpartum mothers interviewed, 225 (59.1%) received information about essential newborn care. Knowledge-wise, 239 (62.7%) participants showed good knowledge, while 142 (37.3%) showed poor knowledge. Of the attitudes, 220 (57.8%) participants had a positive attitude and 161 (42.2%) participants had a negative attitude. Regarding practices, 150 (39.4%) participants had good practices and 231 (60.6%) had bad practices.

**Conclusion:**

there appears to be a significant difference between the perceived knowledge of study participants and the actual practice of neonatal care. Therefore, it is recommended that health professionals provide ongoing postpartum education and training to mothers who have given birth to bridge this gap and bring about meaningful change.

## Introduction

The puerperal period begins when the placenta is expelled and lasts approximately six weeks. A neonate, i.e. child less than 28 days, has the highest risk of death during this critical period. To solve this urgent problem, the World Health Organization (WHO) developed the Essential newborn care (ENC) concept, which provides comprehensive recommendations to improve the newborn´s health. These recommendations include interventions before conception, during pregnancy, immediately after birth and postpartum [1]. The well-being and survival of newborns depend largely on their care. However, neonatal care often does not receive enough attention, even though it plays a crucial role in reducing infant mortality [2].

Newborn care is influenced by mothers' home practices and health services for both mothers and newborns [3]. To reduce the mortality rate in newborns and to improve the practice of immediate help to newborns, it is important to introduce special measures and practices in health services. This can be achieved by upgrading the knowledge and skills of caregivers of newborns during and after delivery [4,5]. Globally, the under-five mortality rate in 2021 was 38 deaths per 1,000 live births, with a significant proportion due to birth complications. In Africa, approximately 300,000 newborns die each year shortly after birth due to inadequate maternal and newborn care. This figure is even higher in sub-Saharan Africa with 74 deaths per 1000 live births recorded [6-9].

According to a report published by the World Health Organization (WHO) in 2015, there were a total of 195,504 deaths among children under the age of five in Ethiopia. Of these deaths, 84,437 were related to childbirth complications. These alarming statistics reveal a significant number of newborn deaths in the home environment, where newborns do not receive the necessary care [10]. The lack of knowledge of traditional birth attendants, combined with deeply rooted cultural beliefs, profoundly affects newborn survival. Immediate care practices provided by primary care providers are central to determining neonatal mortality and morbidity [11,12].

In addition, the health of newborns has been largely neglected, despite significant mortality in health services. This neglect can be due to various factors such as lack of knowledge, unhygienic delivery practices that lead to infections in newborns, bathing the baby immediately after birth, improper care of the umbilical cord, delay in contact between mother and newborn due to the belief that the newborn is dirty and must be cleaned before contact and the underreporting of newborn deaths [13].

However, a significant portion of these deaths are preventable through proven and cost-effective measures. These interventions include improving antenatal care (ANC), ensuring the presence of skilled birth attendants during delivery, and postnatal care (PNC). In addition, although home newborn care packages (HBNC) may not be widely known in our context, they have been effective in treating the main causes of newborn deaths, resulting in mortality reductions of up to 70% [14,15].

It is important to assess mothers' knowledge, attitudes and practices in caring for newborn babies. This is because mothers are usually the primary caregivers of newborns, and their knowledge and practices significantly influence the future of the newborn [16]. Currently, there is little information on knowledge, attitudes and practices related to the essential care of newborns in the Ilubabor zone. Therefore, understanding newborn care processes at the facility level is important to ensure infant survival. This study aims to investigate the latest information on the precise knowledge, attitudes and practices of mothers in essential newborn care by adding additional variables not considered in previous studies.

## Methods

**Study area and period:** the study was conducted from June 20^th^ to August 20^th^ 2023, in public health facilities located in the Illubabor zone of southwestern Ethiopia. Illubabor is one of the 20 zonal governments of the Oromia Regional State, located approximately 600 km west of Addis Ababa, the capital of Ethiopia. In 2020, the total population of the zone government was recorded as 968,303. The zone includes 14 rural, 1 city administration, 23 urban kebeles and 263 rural kebeles. The zone also has adequate health infrastructure, including 2 hospitals, 29 health centers and 263 health posts, with 1,114 health workers from various professional groups and 606 support staff.

**Study design and population:** a mixed cross-sectional descriptive study conducted in institutional settings was utilized for this research. The source populations consisted of all puerperal mothers who gave birth at public health facilities in the Ilubabor zone. The study populations included immediate puerperal mothers who delivered at the designated health facilities during the data collection period that fulfilled the inclusion criteria.

**Sample size determination:** sample size was calculated using the single population proportion formula. The prevalence of good practice in newborn care among postnatal mothers in the north of Shewa zone was 62% [17]. Hence, the sample size was calculated by using the following formula:


$$N=\frac{za/2^{2}p(1-p)}{d2}$$

Where: Z =1.96 confidence limit or critical value at 95% CI P= Proportion d= desired precision estimate. Hence, N = 362, considering a 5% non-response rate; the final sample size was 381. For the qualitative part of the study, in-depth interviews (IDI) with 16 mothers in all health facilities were involved depending on idea saturation.

**Inclusion criteria:** all postnatal mothers who gave birth to neonates alive at selected public health facilities of the Ilubabor zone during the study period were included.

**Exclusion criteria:** mothers who were severely ill and had mental problems (postpartum depression, postpartum blues, and postpartum psychosis) to provide relevant information were excluded from the study.

**Sampling technique:** a multistage sampling method was used to select healthcare facilities and study participants. Initially, 30% of health facilities (two hospitals and six health centers) were randomly selected in the first stage. After that, the unit to be researched was determined using a systematic random sampling technique. The K-value was calculated as the average of births in the previous three months of the data recorded in the selected health facilities, resulting in N/n 640/381=2. A third mother was randomly selected to choose the first mother and skipping all 2 mothers, all participants who met the inclusion criteria were included until the final minimum sample size was reached.

**Data collection tool and data quality control:** the quantitative part of the study used a semi-structured questionnaire filled with face-to-face interviews. The questionnaire was developed based on a review of the relevant literature [2,18-26]. Originally in English, it was translated into the local language, Afan Oromo, and then translated back into English for consistency. Data were collected by eight midwifery professionals who acted as data collectors and four master´s holders who acted as supervisors. To ensure the consistency of variables, the questionnaire was pre-tested among 20 women (5% of the sample size) at Bedele General Hospital located outside the study area. Necessary adjustments or changes were made to the questionnaire based on the results obtained from the pre-test. The internal reliability of the questionnaire was evaluated using Cronbach's alpha, giving a value of 0.86, indicating validity and consistency.

The questionnaire consists of five parts. They are; socio-demographic characteristics, awareness of ENC, knowledge assessment questions, attitude assessment questions and practical question assessment. There are 14 points in the knowledge assessment section; five points for attitude evaluation and 9 points for practice. Before starting data collection, two consecutive training days were organized for both data collectors and supervisors. This training covered the study objectives, the importance of confidentiality and appropriate data collection methods. We got timely feedback from the supervisors after checking the data for completeness and consistency during the data collection period since completed questionnaires were collected daily. Regular monitoring was done to ensure the quality of the data collection process.

In the qualitative phase of the study, sixteen women (two from each facility) participated in individual in-depth interviews (IDIs) lasting an average of 30 minutes. The interview guides were prepared in English and translated into the local language, Afan Oromo. The collected data were analyzed using the Atlas.ti.7.1 software. The in-depth interview (IDI) was conducted using self-contained guides developed through a literature search and collected by experts in qualitative research. Audio recordings were transcribed verbatim and translated into English to expedite the analysis process. Transcripts were thoroughly read and checked several times to ensure a comprehensive understanding of the data. After a line-by-line inductive coding process performed independently by the principal researcher, the focus shifted to those transcripts that contained the most comprehensive information related to the research questions. Once coders achieved consistency, a codebook manual was developed. To ensure accuracy, the principal investigator carefully coded all data. To confirm the interpretation, the author reviewed the document and presented a summary of its contents to a sample of study groups.

**Data processing and analysis:** the collected data were double-checked for accuracy. The data was then input into Excel and analyzed using SPSS version 20. Accordingly, descriptive statistics such as frequency, proportions, mean, and standard deviation were calculated. Tables and narration were used to describe the outcome. The qualitative data was analyzed using the software tool Atlas.ti.7.1.and narration was used to describe the outcome.

**Operational definition:** the puerperal (or postnatal) period begins after delivery and is typically considered to last for six weeks (1).

***Immediate newborn care:*** it is the care given to the neonatal following birth like providing warmth and prevention of heat loss, initiation of breastfeeding, protection from infections, safe umbilical cord tie and cutting and postponing bathing for 24 hours (2).

***Knowledge:*** factual information that the respondents understand about newborn care.

***Good knowledge:*** those who answered greater than 60% of the questions out of the total knowledge-related questions (2).

***Poor knowledge:*** those who answered less than 60% of the question out of the total knowledge-related questions (2).

***Attitude:*** the feeling and beliefs of respondents concerning newborn care.

***Positive attitude:*** those who answered positively greater than 60% of the attitude-related questions (2).

***Negative attitude:*** those who answered positively less than 60% of attitude-related questions (2).

***Practice:*** the intended actions that the respondents have done or do to give care for the newborn baby (2).

***Good practice:*** score greater than 60% on the overall practice question (2)

***Poor practice:*** those who answered less than 60% of the practice-related questions (2).

**Availability of data and materials:** the data sets used and/or analyzed during the current study are available from the corresponding author on reasonable request. The STROBE checklist guideline was used.

**Declaration/Ethical consideration:** the Institutional Research Ethical Review board of Mattu University College of Health Science provided ethical clearance by Ref.No.MDD/146/2016. The endorsement letter was acquired from the health department of Illubabor zone. The collection of data was done in a manner that ensured anonymity, and the investigators were responsible for safeguarding the information. All procedures adhered to the guidelines and regulations outlined in the Declaration of Helsinki. The research objectives were clearly communicated to every puerperal mother, and subsequently, written informed consent was obtained from all participants in the study. No personal information was recorded, and unique codes were assigned to each questionnaire.

## Results

**Socio-demographic characteristics of the study participants:** the study involved a total of 381 puerperal mothers and resulted in a 100% response rate. Of the 381 participants, 222 (58.3%) were aged 25-34. The majority of participants (365 or 95.8%) were married, while 60 (16.35%) reported being unable to read and write (Table 1).

**Table 1:** socio-demographic characteristics of puerperal mothers at public health facilities of Ilubabor zone, 2023 (n=381)

Characteristics (n = 381)	Frequency	Percentage (%)
**Mothers' age**		
15-24	97	25.5
25-34	222	58.3
35-44	62	16.2
Above 45	0	-
**Marital status**		
Single	9	2.4
Married	365	95.8
Divorce	6	1.6
Widow	1	0.26
**Level of education**		
Cannot read and write	60	15.7
Attended primary school	68	17.8
Attended secondary school	192	50.4
Diploma	44	11.5
BA/BSc	15	3.9
MA/MSc	2	0.5
**Mothers' religion**		
Orthodox	119	31.2
Islam	145	38.1
Protestant	115	30.2
Catholic	0	-
Adventist	2	0.52
**Occupation**		
Government employee	102	26.8
Merchant	47	12.3
Farmer	51	13.4
Housewife	155	40.7
Daily laborer	26	6.8
**Living address**		
Urban	214	56.2
Rural	167	43.8
**Ethnicity**		
Oromo	293	76.9
Amahara	63	16.5
Tigre	21	5.5
Others	4	1.1

**Awareness of mothers towards essential newborn care:** among the interviewed puerperal mothers, a significant number of 225 (59.1%) had been provided with diverse information regarding essential newborn care. The majority, comprising 238 (62.5%) of these mothers, received this crucial information from midwives (Table 2).

**Table 2:** awareness of puerperal mothers at public health facilities of Ilubabor zone about essential newborn care and potential source of information, 2023 (n=381)

Item/question	frequency		Percentage %
Are you aware of essential/immediate new born care?	Yes	225	59.1
	No	156	40.9
**Source of information about new born care**			
Doctors	37		9
Midwifery	238		62.6
Family members	48		12.7
Media (radio, TV, broachers etc.)	25		7
Peers/friends	33		8.7
**Type of information received**			
Breastfeeding	198		51.9
Cord care	170		44.6
Eye care	80		20.99
Thermoregulation	116		30.4
Immunization	240		63

**Puerperal mothers knowledge and practice of essential newborn care:** the significance of maintaining a newborn baby's warmth at birth was acknowledged by the majority of participants in the study, precisely 294 individuals (77.2%). However, 87 (22.8%) respondents indicated that they were unaware of this issue. In terms of techniques for providing warmth to a newborn immediately after birth, 291 (76.4%) out of 381 respondents suggested that wrapping the baby in a clean cloth was the appropriate method. Regarding the timing of the first bathing of a newborn, 233 (61.2%) respondents believed that it should take place after one day (24 hours). Conversely, 105 (27.6%) and 43 (11.3%) out of the 381 respondents stated that the newborn should be bathed after six hours and immediately after birth, respectively. In conclusion, among total participants 239 (62.7%) of the women have good knowledge towards essential newborn care whereas 142 (37.3%) of them had poor knowledge.

**Figure 1:**
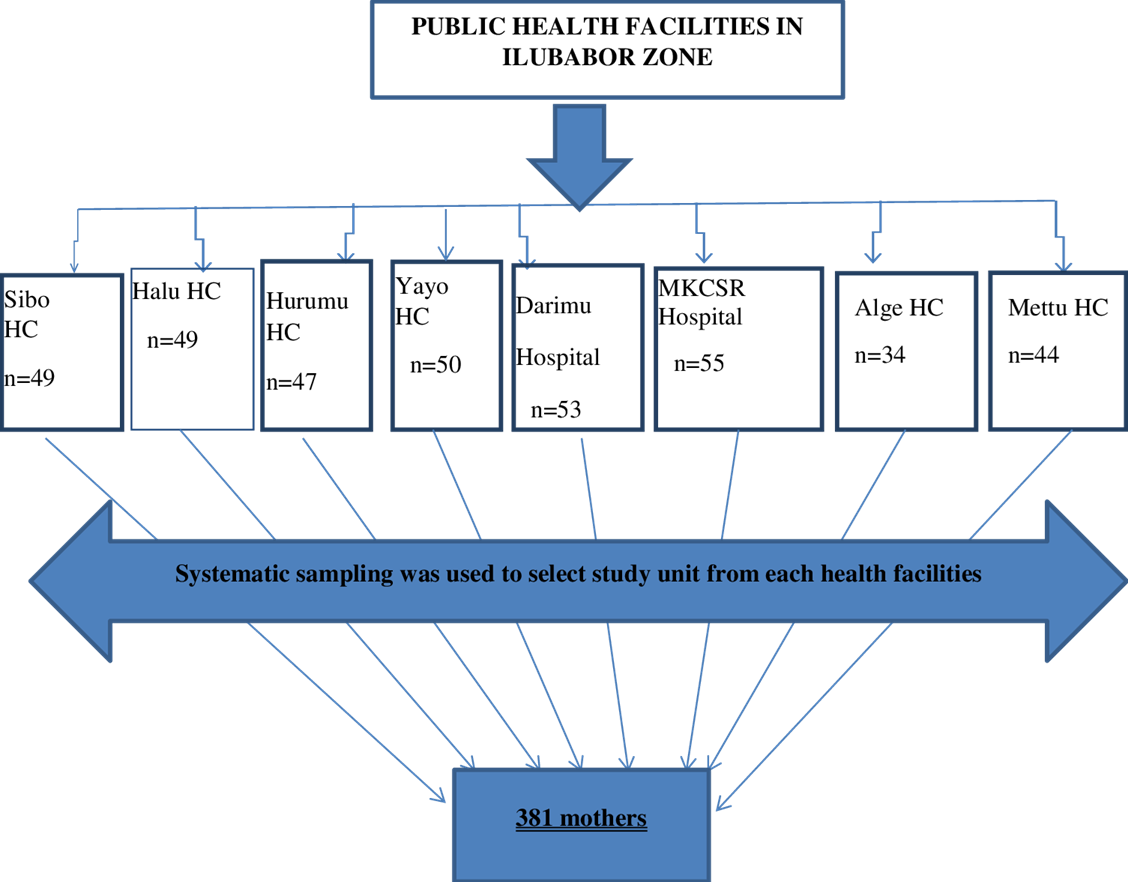
sampling procedure among puerperal mothers at public health facilities of Ilubabor zone, southwest Ethiopia, 2023

**Figure 2:**
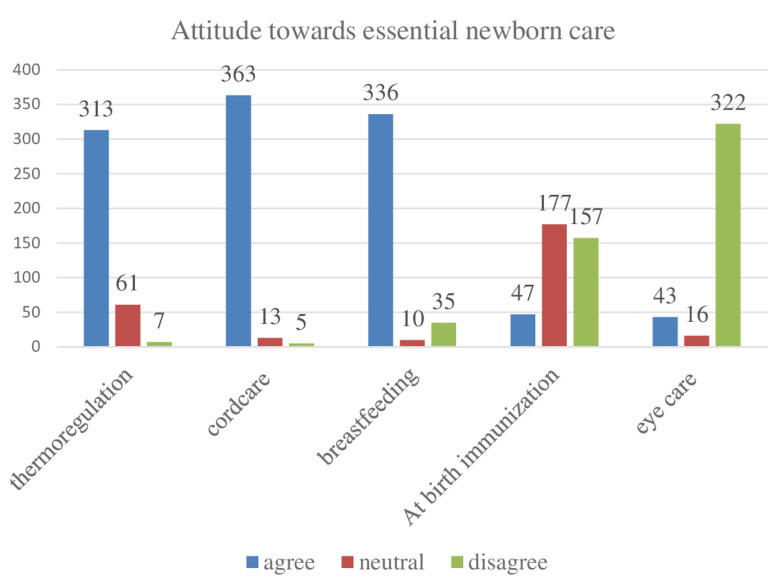
attitude of puerperal mothers towards essential newborn care at public health facilities of Ilubabor zone, southwest Ethiopia, 2023 (n=381)

**Attitude puerperal mothers towards essential newborn care:** among the 381 individuals who participated in the study, a significant majority of 313 respondents (82.2%) agreed that, those infants with low birth weight experience a faster loss of heat compared to babies with normal weight. Conversely, a smaller proportion of participants, specifically 61 individuals, expressed a neutral stance on this matter, while 7 respondents did not provide a clear position (Figure 1). Analyzing the overall data, it is evident that only 220 participants (57.8%) displayed a positive attitude, whereas 161 individuals (42.2%) exhibited a negative attitude towards this issue (Figure 2)

**Puerperal mothers practices of essential newborn care:** among the 381 participants in the study, 219 individuals (57.5%) reported wrapping the newborn baby between 5-10 minutes after delivery, whereas 113 respondents (29.7%) stated that they wrapped the newborn baby in less than 5 minutes after delivery. Overall, out of the 381 women who took part in the study, 150 participants (39.4%) demonstrated good practice, while 231 individuals (60.6%) exhibited poor practice (Table 3).

**Table 3:** practices of puerperal mothers towards essential newborn care at public health facilities of Ilubabor zone, southwest Ethiopia, 2023 (n=381)

Mothers' practices	Frequency	Percentage %
**Thermoregulation**		
**How long after birth did you wrap your baby?**		
In less than 5minutes	113	29.7
5-10 minutes	219	57.5
More than 10 minutes	49	12.8
**How long after birth did you bath your baby?**		
Soon after delivery	30	7.8
1-6 hours after delivery	43	11.3
After 24 hours	308	80.8
**Cleanliness and Cord care**		
**Which instrument did you use to cut cord of your baby?**		
New blade	367	96.3
With used blade if it is sharp	14	3.7
**Which material was used to tie a cord of your baby?**		
Thread	-	-
Cord tie	151	39.6
Cord clamp	230	60.4
**Which one was applied to the cord of your baby?**		
Nothing	222	57.8
Butter/oil	-	-
Chlorhexidine gel	159	41.7
**Breastfeeding**		
**When did start breastfeeding after delivery?**		
Within the first 1 hour	236	61.9
From 2-8 hours	27	7.1
I don't remember	118	30.9
**At what instances did you breastfeed your newborn baby?**		
As often as a baby needs it	197	51.7
Every hour	144	37.8
Every two hours	40	10.5

The quantitative findings were further complemented by the qualitative findings, indicating that there are still issues regarding the implementation of certain essential newborn care practices. The qualitative component specifically examined the areas of cord care, thermal care, and breastfeeding.

The 29-year-old mother reported that "she delivered her child 18 hours ago with the assistance of her mother at home. She was admitted to the hospital because the placenta was not delivered. Following the delivery, the mother bathed the newborn baby with warm water. She mentioned that this practice is common among women in their community. The mother expressed concern that the newborn was exposed to contaminated blood, which is why they opted for immediate bathing".

Out of 381 puerperal mothers, huge number (367 (96.3%)) were stated that new blade should be used to cut the umbilical cord. According to the opinions of most of the respondents *"In the qualitative findings, mothers commonly reported the practice of applying fresh butter or oily substances on and around the stump, regardless of whether delivery took place at home or in a health facility. A 21-year-old mother shared her experience of delivering her first newborn at home with the assistance of her mother. Following delivery, the mother tied the cord with a thread and cut it using a new blade purchased from the market. She mentioned that her mother measured the distance from the baby's abdomen using her fingers to determine where to cut the cord. If the cord was tied and cut one finger's length away from the newborn's abdomen, it would drop within one day; if it was two fingers away, it would drop within two days. Additionally, the mother mentioned that she applied fresh butter on the stump shortly after delivery to prevent dryness and keeps it soft, continuing this practice until the cord eventually fell off*."

Around 236 (61.9%) of the neonate of respondents initiated breastfeeding within one hour after delivery. A 37-year-old interview mother said that "I had no prenatal care follow-up. And I was delivered at here with the help of doctors. After delivery the attendants gave me coffee and Mirinda (soft drink/beverage) before starting breastfeeding, they consider this would be expresses mother breast milk and help newborns to initiate breastfeeding. Then my assistant informed me to stay for some time to breastfeed the neonate".

## Discussion

The study found that most of the participants had sufficient knowledge about the necessary care of newborns, which is consistent with a study conducted in Nepal [27]. In contrast, a study in Udupi district of India found that majority of mothers had a good understanding of newborn care [28]. This difference can be attributed to differences in educational background and different levels of education of mothers about newborn health, which is an important method of disseminating accurate and relevant information.

Regarding specific aspects of newborn health care, mothers showed sufficient knowledge about immediate newborn care. A large number of mothers had knowledge of at least one aspect of basic newborn care. This percentage was higher than the results of a similar study in Ethiopia, where 80.4% of mothers knew at least one aspect of immediate newborn care [29]. These differences may be due to differences in sample size, study duration, and methodology. About 50% of the mothers who participated in this study knew that breastfeeding should start within one hour after birth, which is confirmed by the qualitative aspect of the study. This finding is in contrast to a study in Kenya where about 72% of mothers knew that breastfeeding should start within the first 30 minutes of the baby's life [30]. Mothers in this study showed limited knowledge about the duration of breastfeeding until weaning. However, an overwhelming majority (96.3%) of the respondents were well-informed about the appropriate instrument for cutting a baby's cord, which aligns with a study conducted in Iran where 82.3% of mothers were aware of using a new blade for this purpose [31].

Less than half or 41.7% of the mothers were aware of the use of chlorhexidine gel as the most effective agent for cleaning a baby's cord. In contrast, a study conducted in the upper Himalayas found that a higher percentage (81.8%) of the mothers were aware of this [32]. This difference could be attributed to various factors such as the unavailability of drugs (in Ilubabor zone health facilities, routine use of chlorhexidine was started recently), the study setting, design, and duration, as well as socio-demographic characteristics.

In general, the prevalence of essential newborn care practices was 39.4% [33]. This is similar to the findings of a study conducted in Gurage zone [34], Chencha district of Ethiopia (38.4%), and Nekemte city, western Ethiopia (44.1%) [35], as well as northwest Ethiopia (40.6%). Specifically, less than half of the mothers (29.7%) in this study believed that a baby should be wrapped within 5-10 minutes after delivery, as indicated by the qualitative part of the study. This percentage was slightly lower than the findings of a study conducted in North Ethiopia, where 66.9% of the mothers mentioned the need for immediate wrapping of a baby within 5 minutes of delivery [33]. This difference could be attributed to differences in literacy levels among women and socio-cultural variations.

In this study, 61.9% of the mothers were found to initiate breastfeeding within one hour of delivery as evidenced by qualitative part of the questions. This percentage was lower than the results of a district in Tigray, northern Ethiopia, where 93.2% of mothers started breastfeeding within 30 minutes. The strengths of this study are that it used primary data, with qualitative analysis adding information that was not considered in other studies. However, the study had some limitations. First, due to lack of budget, the study did not examine relevant factors that may affect KAP and ENC. Second, due to the qualitative nature of the analysis conducted in the study, there may be incomplete data and other limitations.

## Conclusion

The results of the study showed that the participants had sufficient knowledge and positive attitudes towards essential newborn care. However, compared to previous studies, the implementation of newborn care did not meet expectations. It is recommended that Ilubabor Health Office and Mattu City Government collaborate with Mattu University to address the knowledge, attitude and practice gaps highlighted in the current study. Health care providers should provide essential newborn care information to expectant mothers at various health facilities that offer prenatal care, delivery services, and newborn and postpartum clinics, even before pregnancy. In addition, public health authorities, especially health assistants, should conduct community health promotion activities on essential maternal newborn care to improve maternal attitudes and newborn care practices. Finally, the dissemination of influential media messages from health authorities through radio and television platforms can significantly contribute to improving mothers' attitudes and practices towards essential newborn care.

### What is known about this topic

- The neonate, which refers to a child under 28 days old, is at the highest risk of mortality during this critical time;
- Ethiopia is one of the countries in which large number of neonatal mortality is recorded among sub-Saharan Africa according to Ethiopian Demographic Health Survey of 2019.

### What this study adds

- Levels of knowledge and attitude towards essential newborn care among puerperal mothers are identified;
- In the body of knowledge, this study addressed the knowledge of the mothers towards essential newborn care using 14 items, i.e. the exact level of knowledge towards essential newborn care including its steps was clearly known in a way that enhance generalization in southwest Ethiopia;
- The exact figure of practice towards essential newborn care among puerperal mothers was demonstrated.
